# Clinical application value of expanded carrier screening in the population of childbearing age

**DOI:** 10.1186/s40001-023-01112-8

**Published:** 2023-04-08

**Authors:** Yuqin Fang, Jingran Li, Miaomiao Zhang, Yuan Cheng, Chaohong Wang, Jiansheng Zhu

**Affiliations:** 1 Affiliated Maternity and Child Health Hospital of Anhui Medical University, Hefei, China; 2 Maternity and Child Health Hospital of Anhui Province, Hefei, China

**Keywords:** Expanded carrier screening, Genetic disease, Gene, Variant

## Abstract

**Objective:**

The objective of this study was to explore the clinical utility of the implementation of expanded carrier screening (ECS) in Chinese population of childbearing age.

**Materials and methods:**

Based on capillary electrophoresis, a first-generation sequencing technology, a prospective screening study of carriers of 15 single-gene diseases was carried out in 327 subjects in Anhui Province, including 84 couples and 159 women of childbearing age, the disease carrier rate, types of screened pathogenic genes, and incidence of both partners carrying the same pathogenic genes were summarized and analyzed.

**Results:**

In 320 people with normal phenotypes who underwent ECS for 15 genetic diseases and 7 spouses who underwent targeted gene sequencing, 65 carriers of at least one disease were detected, with a total carrier rate of 20.31% (65/320). Among the 65 carriers, 81.54% (53/65) carried one genetic variant, 16.92% (11/65) carried two genetic variants, and 1.54% (1/65) carried three genetic variants. In this study, the three diseases with the highest carrier rates were hereditary deafness (8.13%, 26/320), Wilson's disease (4.06%, 13/320), and phenylketonuria (3.13%, 10/320). One high-risk couple (1.19%, 1/84) was detected.

**Conclusions:**

It has certain clinical application value to implement ECS in the population of childbearing age in China.

## Background

Single-gene disease is one of the major causes of malformation and disability in children. Although the incidence rates of single-gene diseases are relatively low, the overall incidence rate is more than 1%, and there are no effective drugs or treatment programs for most single-gene diseases. It is possible to reduce the number of children born with birth defects by conducting expanded carrier screening (ECS) before pregnancy or in early pregnancy, screening for autosomal recessive or X-linked genetic disease-related genes in the population with normal phenotypes, and providing genetic counseling and fertility guidance to these individuals [1].

Initial single-gene disease carrier screening programs were carried out for specific populations, starting with the Tay‒Sachs Disease (TSD) carrier screening project launched in the 1970s [2]. TSD is an autosomal recessive lysosomal storage disease; in infants, TSD is a destructive neurodegenerative disease that causes death before the age of 3–5 years. TSD is more common in German Jewish individuals [3]. The application of carrier screening has reduced the incidence rate of TSD in the German Jewish population by more than 90% [4], which clearly shows that carrier screening can effectively prevent the birth of affected fetuses. The emergence of high-throughput sequencing technology has greatly improved the efficiency, cycle, and cost of testing so that specific populations and more general populations without a family history can be screened for various diseases before or during pregnancy. Scientific research on ECS has been a very hot topic abroad. For example, a study on ECS in the field of assisted reproduction [5] showed that approximately 5% of couples preparing to undergo assisted reproduction surgery were determined to carry disease-causing gene mutations for the same genetic disease. Two percent of female subjects had a pathogenic mutation for an X-linked genetic disease. The researchers estimated that ECS prevented the birth of 1.25% of children with genetic disorders after assisted reproduction. In recent years, many professional associations have published a number of guidelines and statements on carrier screening. Carrier screening has evolved from being limited to specific races and specific diseases at the beginning to recommending screening for all women with reproductive needs and their spouses [6–10].

Currently, China's research on population carrier screening is in its infancy, and there is no relevant guidance or expert consensus. At present, carrier screening work in China is mainly focused on single diseases [11, 12]. Occasionally, research data are provided in ECS-related reports, such as the study by Zhao [13], which involved ECS for 11 recessive diseases in China, proving its feasibility. In this study, capillary electrophoresis, a first-generation sequencing technology, was used to carry out ECS for 15 genetic diseases in the population of childbearing age in Anhui Province; according to detection results, genetic counseling and fertility guidance were provided to effectively reduce birth defects and explore the clinical application value of implementing ECS in China.

## Materials and methods

### Subjects

From January 2020 to November 2020, 327 subjects with normal phenotypes were recruited from the Maternity and Child Health Hospital of Anhui Province. Before examination, all subjects received professional genetic counseling regarding the scope, purpose, significance, limitations, and residual risks of ECS for the target disease; the subjects were fully informed and provided informed consent. This study included 320 patients undergoing ECS for 15 genetic diseases and 7 patients undergoing targeted gene sequencing, including 84 couples and 159 women of childbearing age. Among these patients, 84 were male (25.69%, 84/327) and 243 were female (74.31%, 243/327), with an average age of 31.43 years (21–49 years). This study was approved by the Ethics Committee of Anhui Maternal and Child Health Hospital. All subjects voluntarily underwent ECS and signed informed consent forms.

### Disease selection

In this study, ECS was conducted for 410 pathogenic variants in 21 genes associated with 15 diseases. The selection of these diseases followed the screening criteria recommended by the American College of Obstetricians and Gynecologists (ACOG) [10]. Except for hereditary deafness, all included diseases were single-gene diseases with a severe prognosis. However, due to the high carrier rate of hereditary deafness in the Chinese population, deafness gene screening can help provide early intervention information to improve the language development of affected children, so it was also included in the study system [14, 15]. The fifteen genetic diseases are hereditary deafness, thalassemia, duchenne muscular dystrophy, hemophilia A, Fragile X syndrome, X-linked ichthyosis, spinal muscular atrophy, phenylketonuria, methylmalonic acidemia, congenital adrenal hyperplasia, Wilson's disease, Xp11.22 microduplication syndrome, Pelizaeus-Merzbacher disease, MECP2 duplication syndrome, and Xq28 microrepeat syndrome mediated by Int22h1/Int22h2.

### Targeted gene sequencing

This study mainly relied on the gold standard technology for clinical gene analysis, capillary electrophoresis, which is a first-generation sequencing technology. A total of 2 ml of peripheral blood was collected from the subjects; DNA was extracted with the DNA extraction kit produced by Beijing Tiangen Biochemical Technology, and then probe connection, multiple PCR, and gene sequencing were conducted. The ABI3730 and ABI3500 first-generation sequencing platforms were used for the precise sequencing and analysis of multiple mutation types (point mutations, deletion duplications, short tandem duplications, inversions, gene fusions, etc.) in the target disease genes. The detection technologies included single-nucleotide polymorphism (SNP) scan technology, copy number variation (CNV) plex technology, AQ-PLP technology, iMLDR technology, special fluorescent PCR, gap-PCR, etc., and the results of all detected gene mutations were verified.

### Screening modes

This study included two screening modes. The first mode was simultaneous screening, in which peripheral blood was collected from 67 couples at the same time for ECS of 15 single-gene diseases. The second mode was sequential screening. In this study, 176 women underwent ECS. Among 176 women, 38 (21.59%, 38/176) were determined to be carriers. After outpatient genetic counseling, it was recommended that the spouses of these positive carriers be recalled for corresponding genetic testing. At present, 17 (44.74%, 17/38) spouses have undergone follow-up genetic testing, of whom 10 have chosen to undergo ECS, and the other 7 have chosen to have the target gene sequenced only for the gene mutation carried by the woman.

### Statistical analysis

The data collected in this study were input into the computer and analyzed with SPSS 23.0 statistical software. This study included 320 patients undergoing ECS for 15 genetic diseases and 7 patients undergoing targeted gene sequencing. We mainly conducted descriptive statistical analysis on 320 subjects receiving ECS, calculated the total carrier rate of the target diseases and the carrier rate of pathogenic genes in the region, and conducted descriptive statistical analysis on the types of pathogenic genetic variants detected in this study.

### Genetic counseling

When a subject was determined to be a carrier of a target disease, their spouse was recalled for appropriate genetic testing. If a pregnant was a carrier of an X-linked genetic disease, the risk of her offspring being a male patient or a female carrier was discussed. If necessary, prenatal diagnosis was provided with full informed consent and at the request of high-risk couples to determine whether the fetus had the disease. In addition, for positive carriers, it was recommended that members of their immediate family who are of suitable age for pregnancy also be screened. In addition, follow-up was conducted for high-risk couples with subsequent pregnancy outcomes and their neonates, including physical examinations, hearing screening, and screening for any other clinical or genetic abnormalities. In the case of abortion, stillbirth or neonatal malformation during pregnancy, fetal tissue or neonatal peripheral blood was collected for detection whenever possible.

## Results

### Overall carrier frequencies

**Fig. 1:**
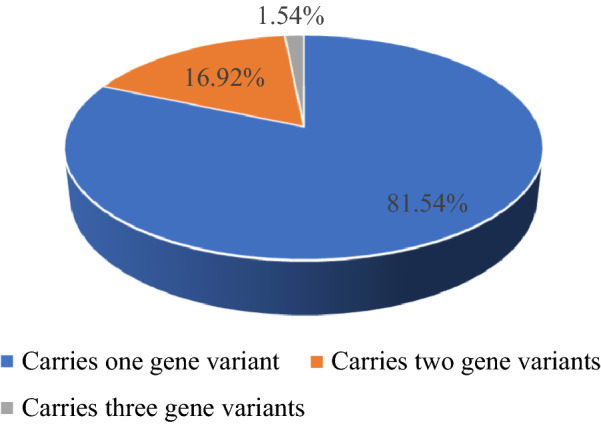
Distribution of the 65 carriers. Among the 65 carriers, 81.54% (53/65) carried one genetic variant, 16.92% (11/65) carried two genetic variants, 1.54% (1/65) carried three genetic variants

**Fig. 2:**
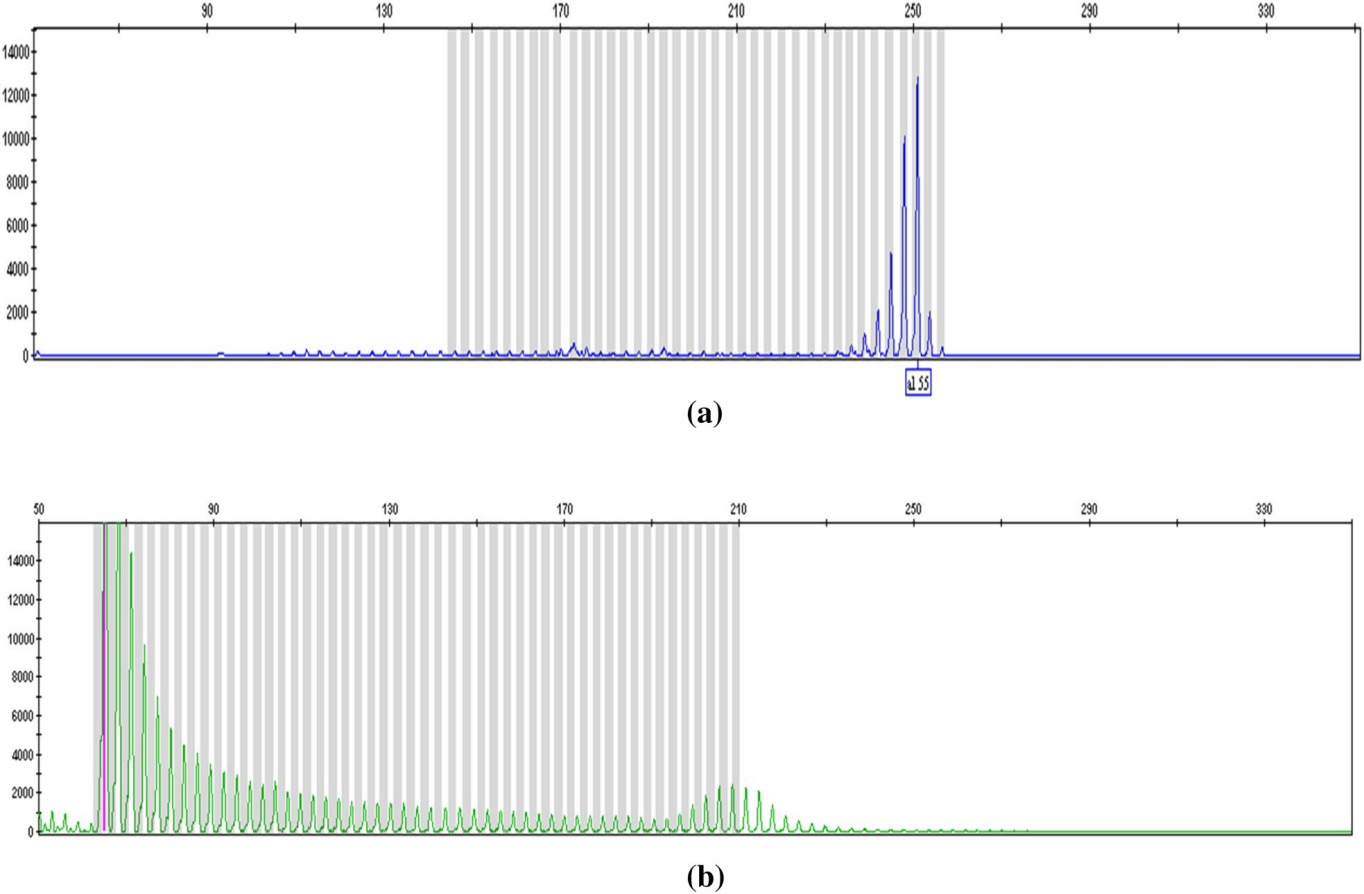
Map of FMR1 gene-specific PCR data in the male premutation carrier of Fragile X syndrome. The fluorescent primer PCR technique was used, which consisted of two reaction systems, namely, specific PCR containing the target region (**a**) and repeat-primed PCR containing CGG repeats (**b**). The data showed that the number of CGG repeats was 55, indicating a hemizygous mutation of FMR1 (NM_002024.5) in Fragile X syndrome. The horizontal coordinate indicates the segment length, and the vertical coordinate indicates the signal strength

Among the 320 subjects who underwent ECS, 65 were identified as carriers of the target diseases, with an overall carrier rate of 20.31% (65/320). Among the 65 carriers, 53 (81.54%, 53/65) carried one genetic variant, 11 (16.92%, 11/65) carried two genetic variants, and 1 (1.54%, 1/65) carried three genetic variants, as shown in Fig. 1. The detected carriers included one (1.54%, 1/65) male premutation carrier of Fragile X syndrome, and the test results are shown in Fig. 2. In this case, the number of FMR1 gene CGG repeats was 55, indicating a premutation type. The incidence of premutations in males in this study population is 1 in 77. The remaining 64 subjects (98.46%, 64/65) were carriers of autosomal recessive diseases.

All genetic variants detected in the study were validated by other methods, such as Sanger sequencing, multiplex ligation-dependent probe amplification, gap-PCR, etc. The results showed that the mutation sites detected in the study were 100% consistent with the verification results.

### The carrying frequencies of each target gene

In this study, a total of 12 subjects were determined to have pathogenic genes for target diseases, of whom 8 had a carrier frequency of more than 1%. The carrier frequencies were as follows: 4.38% (14/320) for the GJB2 gene for hereditary deafness; 3.75% (12/320) for the SLC26A4 gene for hereditary deafness; 4.06% (13/320) for the ATP7B gene for Wilson’s disease; 2.81% (9/320) for the CYP21A2 gene for congenital adrenal hyperplasia; 2.50% (8/320) for the SMN1 gene for spinal muscular atrophy; 2.50% (8/320) for the PAH gene for phenylketonuria; 1.56% (5/320) for the HBA1/HBA2 gene for alpha-thalassemia; 1.25% (4/320) for the MMACHC gene for methylmalonic acidemia; and the remaining four carrier frequencies were less than 1% (Table 1).

**Table 1:** Carrier rates of pathogenic genes for each target disease in the 320 subjects who underwent ECS

Disease	Disease gene	Number of carriers (*n*, case)	Carrier rate (%)
Hereditary deafness	GJB2	14	4.38
	SLC26A4	12	3.75
Wilson's disease	ATP7B	13	4.06
Congenital adrenal hyperplasia	CYP21A2	9	2.81
Spinal muscular atrophy	SMN1	8	2.50
Phenylketonuria	PAH	8	2.50
	PTS	2	0.63
Thalassemia	HBA1/HBA2	5	1.56
	HBB	1	0.31
Methylmalonic acidemia	MMACHC	4	1.25
	MMUT	1	0.31
Fragile X syndrome	FMR1	1	0.31

*n* the number of carriers of the pathogenic genes in the study subjects

### Variation in each target gene

A total of 42 types of pathogenic genetic variants for the target diseases were detected in this study, among which 7 types of variants were detected in more than 2 subjects, accounting for 41.03% (32/78) of all detected variants (Table 2). The top three most common variants were SLC26A4: c.919-2A > G (7 subjects, 1.09%), GJB2: c.235delC (6 subjects, 0.94%), and SMN1: EX7-8DEL (6 subjects, 0.94%).

**Table 2:** 42 types of pathogenic genetic variants detected in this study

Type	Genetic variants	Allele number (*n*, case)	Gene frequency (%)
1	SLC26A4: c.919-2A > G	7	1.09
2	GJB2: c.235delC(p.L79Cfs*3)	6	0.94
3	SMN1: EX7-8DEL	6	0.94
4	HBA1/HBA2: -α3.7	4	0.63
5	ATP7B: c.2333G > T(p.R778L)	3	0.47
6	ATP7B: c.3316G > A(p.V1106I)	3	0.47
7	PAH: c.721C > T(p.R241C)	3	0.47
8	ATP7B: c.2621C > T(p.A874V)	2	0.31
9	ATP7B: c.588C > A(p.D196E)	2	0.31
10	CYP21A2: c.1069C > T(p.R357W)	2	0.31
11	CYP21A2: c.1451_1452delGGinsC(p.R484Pfs*58)	2	0.31
12	CYP21A2: c.293-13A/C > G	2	0.31
13	CYP21A2: CH-8	2	0.31
14	GJB2: c.176_191del(p.G59Afs*18)	2	0.31
15	GJB2: c.299_300delAT(p.H100Rfs*14)	2	0.31
16	GJB2: g.20398370-20523823del	2	0.31
17	PAH: c.728G > A(p.R243Q)	2	0.31
18	SMN1: EX7DEL	2	0.31
19	FMR1: CGG repeats: 55	1	–
20	ATP7B: c.2804C > T(p.T935M)	1	0.16
21	ATP7B: c.2975C > T(p.P992L)	1	0.16
22	ATP7B: c.3089G > A(p.G1030D)	1	0.16
23	CYP21A2: c.518 T > A(p.I173N)	1	0.16
24	GJB2: c.35dupG(p.V13Cfs*35)	1	0.16
25	GJB2: c.416G > A(p.S139N)	1	0.16
26	HBA2: c.369C > G(p.H123Q)	1	0.16
27	HBB: c.217dupA(p.S73Kfs*2)	1	0.16
28	MMACHCc.1A > G(p.M1?)	1	0.16
29	MMACHC: c.482G > A(p.R161Q)	1	0.16
30	MMACHC: c.609G > A(p.W203*)	1	0.16
31	MMACHC: c.80A > G(p.Q27R)	1	0.16
32	MMUT: c.729_730insTT(p.D244Lfs*39)	1	0.16
33	PAH: c.1238G > C(p.R413P)	1	0.16
34	PAH: c.482 T > C(p.F161S)	1	0.16
35	PAH: c.611A > G(p.Y204C)	1	0.16
36	PTS: c.259C > T(p.P87S)	1	0.16
37	PTS: c.286G > A(p.D96N)	1	0.16
38	SLC26A4: c.1229C > T(p.T410M)	1	0.16
39	SLC26A4: c.1520delT(p.L507*)	1	0.16
40	SLC26A4: c.1707 + 5G > A	1	0.16
41	SLC26A4: c.1975G > C(p.V659L)	1	0.16
42	SLC26A4: c.2168A > G(p.H723R)	1	0.16

*n* the number of certain genetic variants detected in this study subjects. %, the proportion of a gene to the total number of alleles in the gene pool of the study subjects

### High-risk couples and their follow-up genetic counseling

Among 84 couples, one (1.19%) was found to be at high risk of having a child with autosomal recessive deafness. In this couple, the woman was first screened as an extensibility carrier for 15 genetic diseases, and the results showed that she was a carrier of the pathogenic genes ATP7B and GJB2 for Wilson’s disease and hereditary deafness, respectively. After genetic counseling, her spouse chose to undergo sequencing for the target genes (GJB2 and ATP7B), namely, GJB2: c.109G > A (p.Val37Ile), was detected, which usually leads to autosomal recessive hereditary deafness. C.109G > A has incomplete dominance. According to the literature [16, 17], GJB2: c.109G > A compound heterozygosity typically results in mild-to-moderate hearing loss; because the woman carried GJB2: c.35dupG (p.V13Cfs * 35), professional and detailed genetic counseling was provided for the couple. Since the couple considered the fetus to be a precious child, they refused prenatal diagnosis and accepted that they might have a deaf child; we followed up on the pregnancy outcome of the pregnant woman and the phenotype of the newborn. The high-risk woman gave birth to a female baby with a normal phenotype by cesarean section. Her newborn had normal physical examination results and passed the hearing screening. This family will be followed up in the future.

## Discussion

Single-gene recessive diseases include autosomal recessive and X-linked recessive genetic diseases, which usually have no family genetic history and cannot be detected in routine prenatal examination; these diseases are discovered after birth when symptoms slowly appear. Recessive genetic diseases easily cause blindness, deafness, body deformities, intellectual disability, growth retardation, the dysfunction or failure of tissues and organs, and even serious harm, such as death, which brings a heavy burden to families and society. In 2011, for the first time, Bell et al. [18] used next-generation sequencing technology to screen 104 subjects for 448 recessive genetic diseases and found that each person carried 2.8 pathogenic mutation genes, highlighting the need for ECS in the population. ECS provides screening for a variety of autosomal recessive and X-linked genetic diseases. With the widespread development of carrier screening and the development of gene testing technology, the reproductive decision-making of couples can be better guided, and the risk of serious single-gene diseases in offspring can be effectively reduced [19]. Therefore, capillary electrophoresis, a first-generation sequencing technology, was used in this study to screen for single-gene disease carriers in the population of childbearing age with normal phenotypes in Anhui Province, which is conducive to providing timely intervention measures for high-risk couples before pregnancy or in early pregnancy and reducing birth defects.

The total carrier rate of pathogenic gene variations in the population screened was 20.31% in this study, indicating the need for carrier screening. Lazarin et al. [20] conducted panethnic carrier screening for 108 diseases, and the total carrier rate was 24%. Zhao et al. [13] performed carrier screening for 11 diseases in a Chinese multiethnic population, with a total carrier rate of 27.49%. The possible reasons for the differences in the results are related to the differences in the regions of the studied populations, sample sizes, types of diseases screened, and sequencing technology used. Among the 65 carriers detected, 2 carried two different mutations of the ATP7B gene, namely, ATP7B: c.588C > A (p.D196E) and ATP7B: c.3316G > A (p.V1106I). Because the phenotypes of the subjects were normal, according to the literature [21], the c.588C > A mutation of ATP7B is usually linked with the c.3316G > A mutation. In this state, it was estimated that the tested person was a carrier of hepatolenticular degeneration. The top three pathogenic genes in this study were GJB2, ATP7B, and SLC26A4; 14 GJB2 carriers were detected, and the most common mutation site was c.235delC (p.L79Cfs*3). A total of 12 SLC26A4 carriers were detected, and the most common mutation site was c.919-2A > G; a total of 13 ATP7B carriers were detected, and the most common mutations were c.2333G > T (p.R778L) and c.3316G > A (p.V1106I). The common mutation spectrum of GJB2 and SLC26A4 in this study was consistent with previous literature reports of the Chinese population [22, 23], while the mutation spectrum of ATP7B was inconsistent with a research report of Chinese patients with Wilson's disease [24]. In addition, we identified a male premutation carrier of Fragile X syndrome. The CGG repeats of male premutation carriers are not expanded when they are transmitted to the next generation, so male premutation carriers generally do not have an increased risk of fetal disease. However, if a daughter is born, she will be a premutation carrier, and genetic counseling is recommended [25, 26]. Among the 84 couples, 1 (1.19%) was found to be a high-risk couple, with both partners being carriers of type 1A (GJB2 gene) autosomal recessive deafness, and their risk of giving birth to deaf children was 25%. For high-risk couples, genetic counseling is needed to help the partners choose prenatal diagnosis or delivery management to improve clinical outcomes. The Chinese population has a high carrier rate of the deafness gene. A multicenter study by Cai on genetic screening for hereditary deafness among newborns in Zhejiang Province, China showed that 8.71% of newborns carried at least one hereditary deafness-related variant [27]. The carrying rate of hereditary deafness in this study was 8.13%. Therefore, genetic deafness diseases were included for screening in this study, and high-risk childbearing couples were identified. Genetic disease screening, which is conducive to early detection and diagnosis, can effectively avoid the occurrence of deafness and birth defects and allows early intervention for newborns, which is conducive to improving language development.

With the development of sequencing technology, next-generation sequencing (NGS) has been widely used in carrier screening for single-gene diseases with the advantages of high throughput and low cost [28]. However, NGS technology still has limitations in genetic screening for single-gene diseases. First, it cannot detect some special gene mutations with a high incidence of genetic diseases (such as pseudogenes, inversions, and CGG repeat mutations). Second, positive NGS test results still need to be verified by generation sequencing. In addition, the most important thing is that NGS technology has a high probability of detecting mutations of unknown clinical significance, and it is difficult for laboratories to interpret these test results in an accurate and detailed way, causing great confusion and burdens for clinicians and patients [28]. Therefore, in this study, carrier screening was performed by capillary electrophoresis-based multiple PCR analysis, which could screen for some special high incidence genetic diseases that could not be detected by NGS, including congenital adrenal hyperplasia (CAH), spinal muscular atrophy (SMA), and Fragile X syndrome. In this study, the carrier rates of CAH and SMA were 2.81% and 2.50%, respectively, suggesting that screening for these two diseases is warranted.

However, this study also has some limitations. First, a limited number of definite pathogenic sites were included, and pathogenic mutations and emerging variants outside the detection range could not be identified, which may increase residual risks. The pretest and posttest consultations to inform the subjects of the benefits and limitations of this study needs to be emphasized. Second, we observed 8 out of the 15 genetic diseases in the study population, which may be related to the small sample size and the types of diseases screened. We will include more subjects for screening in the future. Third, due to time constraints and insufficient follow-up, the follow-up of pregnancy outcomes and newborns will be carried out in the future. Finally, the results of this study were not evaluated by NGS. In the future, we will continue to conduct in-depth research to compare the results of ECS in this study with those of NGS and explore the consistency of the two methods in estimating overlapping genetic variants.

## Conclusion

In conclusion, this study provides an inexpensive and effective method for ECS, and the implementation of expanded carrier screening in China has certain clinical application value and is an important measure to prevent birth defects.

## Data Availability

The data used and analyzed in this study can be obtained from the corresponding author on reasonable request.

## Abbreviations

ECS: Expanded carrier screening
TSD: Tay‒Sachs disease
ACOG: American College of Obstetricians and Gynecologists
SNP: Single-nucleotide polymorphism
CNV: Copy number variation
NGS: Next-generation sequencing
CAH: Congenital adrenal hyperplasia
SMA: Spinal muscular atrophy
